# Subgroup analyses in confirmatory clinical trials: time to be specific about their purposes

**DOI:** 10.1186/s12874-016-0122-6

**Published:** 2016-02-18

**Authors:** Julien Tanniou, Ingeborg van der Tweel, Steven Teerenstra, Kit C. B. Roes

**Affiliations:** Julius Center for Health Sciences and Primary Care, UMC Utrecht, Universiteitsweg 100, 3584 CG Utrecht, The Netherlands; College ter Beoordeling van Geneesmiddelen, Dutch Medicines Evaluation Board, Graadt van Roggenweg 500, 3531 AH Utrecht, The Netherlands; Department of Health Evidence, Section Biostatistics, Radboud University Medical Centre, Geert Grooteplein 21, 6525 GA Nijmegen, The Netherlands

**Keywords:** Clinical trials, Subgroups, Subgroup analysis

## Abstract

**Background:**

It is well recognized that treatment effects may not be homogeneous across the study population. Subgroup analyses constitute a fundamental step in the assessment of evidence from confirmatory (Phase III) clinical trials, where conclusions for the overall study population might not hold. Subgroup analyses can have different and distinct purposes, requiring specific design and analysis solutions. It is relevant to evaluate methodological developments in subgroup analyses against these purposes to guide health care professionals and regulators as well as to identify gaps in current methodology.

**Methods:**

We defined four purposes for subgroup analyses: (1) Investigate the consistency of treatment effects across subgroups of clinical importance, (2) Explore the treatment effect across different subgroups within an overall non-significant trial, (3) Evaluate safety profiles limited to one or a few subgroup(s), (4) Establish efficacy in the targeted subgroup when included in a confirmatory testing strategy of a single trial. We reviewed the methodology in line with this “purpose-based” framework. The review covered papers published between January 2005 and April 2015 and aimed to classify them in none, one or more of the aforementioned purposes.

**Results:**

In total 1857 potentially eligible papers were identified. Forty-eight papers were selected and 20 additional relevant papers were identified from their references, leading to 68 papers in total. Nineteen were dedicated to purpose 1, 16 to purpose 4, one to purpose 2 and none to purpose 3. Seven papers were dedicated to more than one purpose, the 25 remaining could not be classified unambiguously. Purposes of the methods were often not specifically indicated, methods for subgroup analysis for safety purposes were almost absent and a multitude of diverse methods were developed for purpose (1).

**Conclusions:**

It is important that researchers developing methodology for subgroup analysis explicitly clarify the objectives of their methods in terms that can be understood from a patient’s, health care provider’s and/or regulator’s perspective. A clear operational definition for consistency of treatment effects across subgroups is lacking, but is needed to improve the usability of subgroup analyses in this setting. Finally, methods to particularly explore benefit-risk systematically across subgroups need more research.

**Electronic supplementary material:**

The online version of this article (doi:10.1186/s12874-016-0122-6) contains supplementary material, which is available to authorized users.

## Background

Confirmatory (Phase III) clinical trials aim to provide conclusive evidence on the efficacy and safety of new drugs, usually as compared to standard treatments. The conclusions from such studies are typically considered applicable to the whole study population. However, in light of growing biological and pharmacological knowledge leading to more personalized medicine and targeted therapies, it is well recognized that the treatment effect of a new drug might not be homogeneous across the study population. Subgroup analyses are therefore essential to interpret the results of clinical trials. If subgroups with a potential worse or better benefit-harm balance exist, identification is fundamental in the interest of patients.

The methodology and reporting of subgroup analyses have been subject of research and several literature reviews have been conducted. According to Pocock et al. [1], 70 % (35/50) of reported trials contained subgroup analyses, of which 60 % (21/35) claimed subgroup differences. The total number of reported subgroup analyses per published trial varied from one to 24 with a median of four. In 20 % of the trials, subgroup analyses were purely descriptive. Thirty-seven % of trials reported *p*-values for treatment effect within subgroups and 43 % (15/35) used tests of interaction. Hernandez et al. [2] selected 63 cardiovascular clinical trials, of which almost two thirds (39/63) reported subgroup analyses. A subgroup effect was claimed in over half of these (21/39) based on either interaction tests (7/11) or subgroup-specific tests (14/28). Wang et al. [3] reviewed 59 clinical trials, of which 34 performed more than five subgroup analyses and five were unclear about the number of subgroup analyses performed. They found that in the majority of papers it was not specified whether subgroup analyses were pre-specified or performed post-hoc (40/59). In Gabler et al. [4], the authors reviewed 319 studies from high-impact factor journals (BMJ, JAMA, NEMJ, The Lancet, Annals of Internal Medicine). They found that 29 % (92/319) of the studies reported interaction analyses, 28 % (88/319) reported subgroups analyses only without formal statistical comparisons and 43 % (139/319) did not report on the heterogeneity of the treatment effect. Sun et al. [5, 6] investigated the impact of industry funding on the reporting of subgroup analyses in randomized controlled trials. Their study included 469 randomized controlled trials published in 2007, of which 207 (44 %) reported subgroup analyses. Subgroup analyses were more frequent in high impact journals, non-surgical trials and trials with larger sample size. When the primary outcome was not significant, industry funded trials were more likely to report subgroup analyses than non-industry funded trials, contrary to when there was a statistically significant primary outcome.

Subgroup analyses are known to be prone to statistical and methodological issues such as inflation of type I error due to multiple testing, low power, inappropriate statistical analyses or lack of pre-specification. To deal with these issues, guidelines for the design, analysis, interpretation and reporting of subgroup analyses have been proposed [7–12]. They generally share the same main points: the number of subgroups to be tested should be small, subgroups of interest should be pre-specified and based on a strong biological reasoning or based on observed effects in the subgroup in previous studies, adjustment for multiple testing should be considered, subgroup-treatment interaction tests should be preferred to subgroup-specific tests, all subgroups tested should be reported including whether they are pre-planned or post hoc.

Despite such guidance, the assessment of subgroup analyses remains complex. For example, the CAPRIE trial aimed to show superiority of clopidogrel to aspirin in the secondary prevention of cardiovascular events in patients at risk of ischemic events. The primary endpoint was the first occurrence of myocardial infarction (MI), ischemic stroke, or vascular death in patients with atherothrombosis. The intent-to-treat analysis showed a relative risk reduction (RRR) of 8.7 % in favor of clopidogrel (*p* = 0.043). In an additional analysis, the CAPRIE investigators showed that heterogeneity was observed (*p* = 0.042) depending on the qualification of prior cardiovascular events, which was used as a stratification factor at randomization: prior MI: RRR = 7.3 %, prior stroke: RRR = −3.7 %, symptomatic peripheral arterial disease: RRR = 23.8 %. This observed heterogeneity led two regulatory agencies to different assessments. The National Institute for Health and Care Excellence (NICE, English and Welsh authority) concluded a clinical benefit for the overall population whereas the Institut für Qualität und Wirtschaftlichkeit im Gesundheitswesen (IQWiG, German authority) concluded efficacy only for the most beneficial subgroup of patients (symptomatic peripheral arterial disease) [13–15]. The disparate conclusion by the two regulatory agencies illustrates the diverging views or interpretations, and the lack of consensus and international standards for the conduct and interpretation of subgroup analyses.

We observed that methodological papers on subgroup analyses sometimes lacked a clear connection between the methodological solutions and specific purposes. Five different purposes for subgroup analyses have been suggested by Grouin et al. [16], in the context of market authorisation of new drugs: (i) to confirm that efficacy benefits observed in the trial are consistently seen across subgroups, (ii) to identify subgroups with larger treatment effect when the study reaches an overall statistically significant effect, (iii) to check specific subgroups that *a priori* are suspected to show less or no treatment effect, (iv) to identify a statistically positive subgroup in case of a non-significant overall effect, and (v) to identify safety problems limited to one or few subgroups. To gain systematic insight in the “state of the art” and areas of research needed, we reviewed the proposed methodological solutions for this “purpose based” framework.

We first explain the search strategy as well as the “purpose based” framework. Next, the main results of the study are presented. We conclude with a critical discussion and suggestions for further methodological development.

## Methods

### Search strategy

This review covers papers published between January 2005 and April 2015. The last search was conducted on PubMed the 01st of May 2015. The search was restricted to statistical and methodological journals, i.e., the “Journal of Biopharmaceutical Statistics”, “Statistics in Medicine”, “BMC Medical Research Methodology”, “Statistical Methods in Medical Research”, “Contemporary Clinical Trials”, “Trials”, “Clinical Trials”, “Pharmaceutical Statistics”, “Drug Information Journal”, “Biostatistics”, “Biometrical Journal”, “Biometrika”, “Statistical Methodology” and “Biometrics”. Various keywords such as “subpopulation”, “subset”, “subgroup” or “interaction” were considered. In addition reference lists of identified papers were checked, without restriction to these journals. The complete algorithm is provided in the Additional file 1.

### Framework for selection and review

Potentially relevant papers were selected on their title and abstract. To structure our review we adapted the five purposes proposed by Grouin et al. [16]. Purpose (i) and (iii), trying to establish consistency either in positive or negative direction for either all clinically important subgroups or a selected set of subgroups, were merged. Purposes (ii) and (iv), identifying favorable subgroup(s) and aiming to exploit heterogeneity, were also merged. Purpose (v), which concerns safety rather than efficacy, was maintained as proposed by Grouin et al. Finally, we added a specific purpose dedicated to confirmatory subgroup strategies. To summarise, four distinct purposes constitute the framework of this study:

1. *Investigate the consistency of treatment effect* across subgroups of clinical importance.
2. *Explore the treatment effect* across different subgroups *within an overall non-significant trial.*
3. *Evaluate safety profiles* limited to one or a few subgroup(s).
4. *Establish efficacy in the targeted subgroup* when included in a *confirmatory testing strategy* of a single trial.

The methodological papers could fall in none, one or more of these categories. The classification of a paper in a category is based either on an explicit statement on the purpose, or was inferred by the first author from the full text.

## Results

**Fig. 1:**
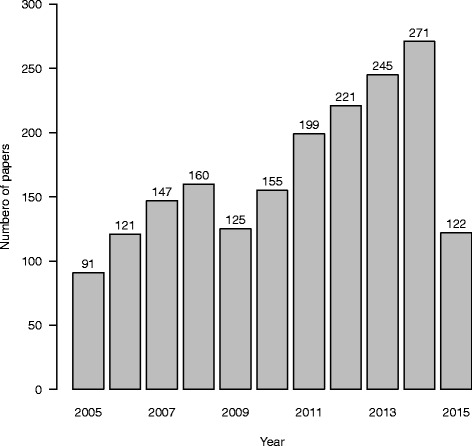
Number of papers published per year

In total 1857 potentially eligible papers were identified. There is a clear increase in the volume of research over time (Fig. 1). This recent activity in the area of subgroup analysis illustrates its importance in drug development.

**Fig. 2:**
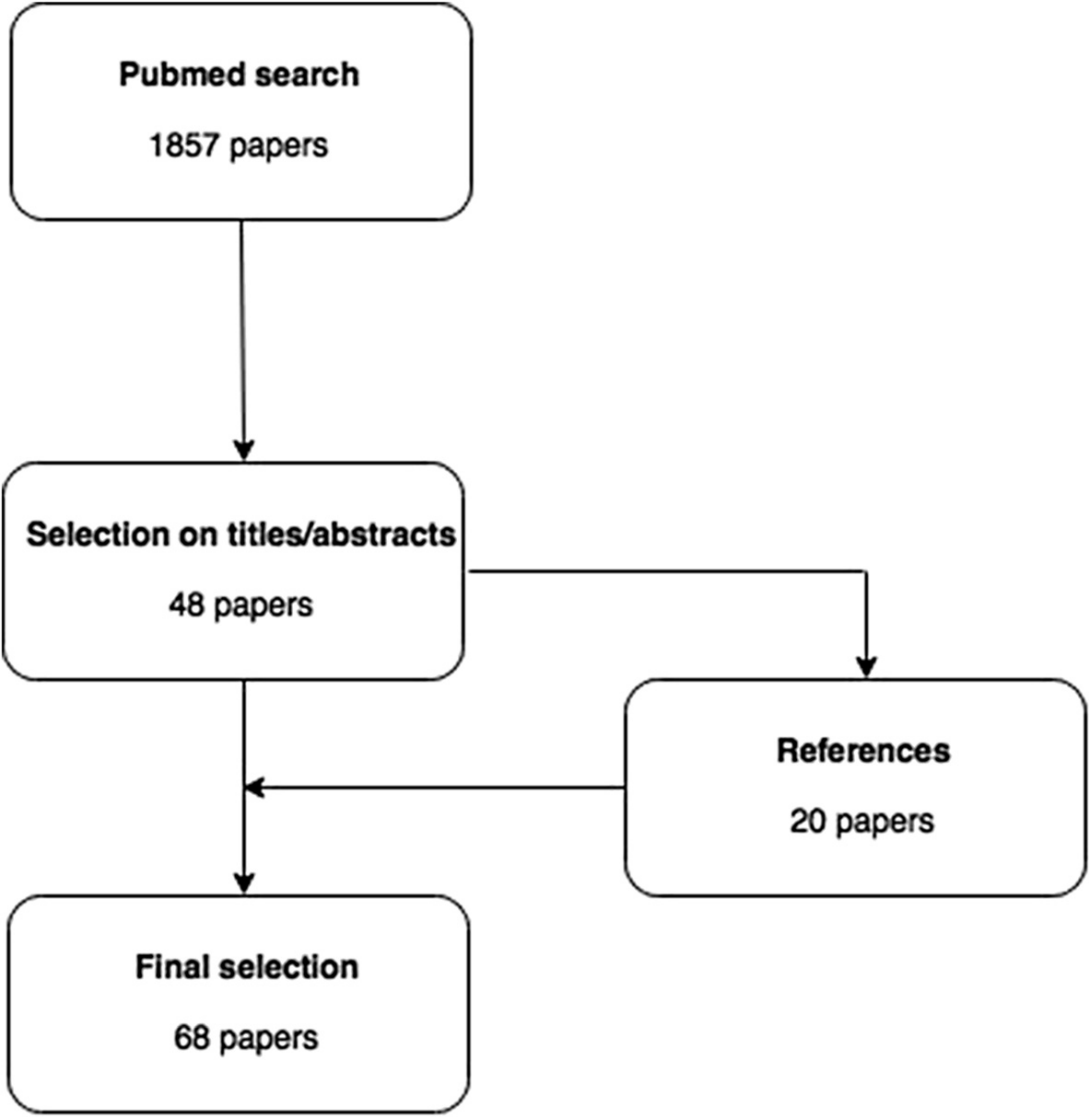
Study flowchart

Forty-eight papers were selected based on the search strategy (Fig. 2). Twenty additional relevant papers were identified from their references, leading to 68 papers in total.

**Fig. 3:**
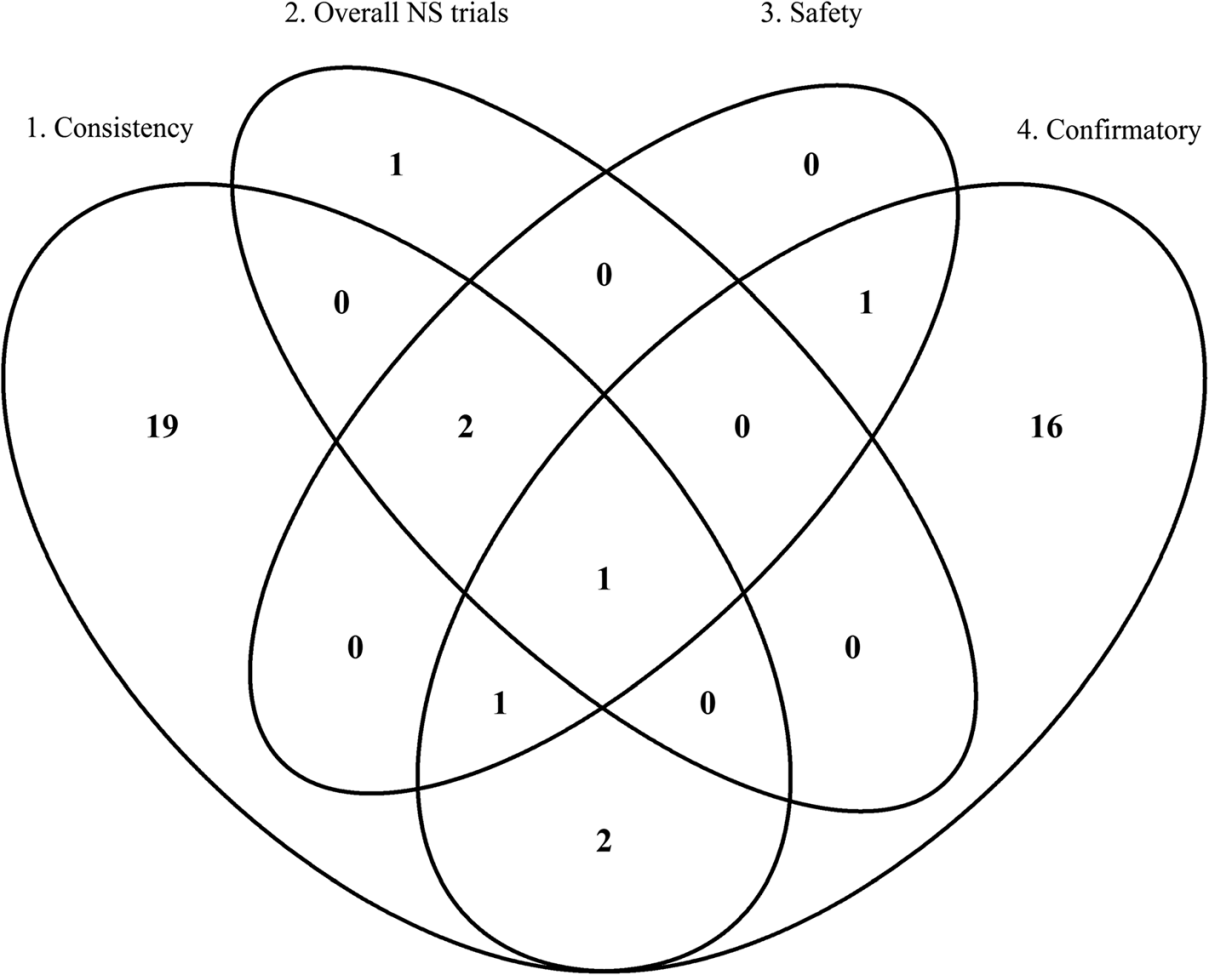
Venn diagram of the classification

The Venn diagram (Fig. 3) shows the classification of papers across purposes. Thirty-six papers were dedicated to exactly one purpose: 19 to purpose 1, 16 to purpose 4 and one to purpose 2. Seven papers were dedicated to more than one purpose. The remaining 25 papers could not be classified to a specific purpose.

Thus, most papers addressed purpose 1, “*Investigate the consistency of treatment effect across subgroups of clinical importance*”(25/68) or purpose 4, “*Establish efficacy in the targeted subgroup when included in a confirmatory testing strategy of a single trial”* (21/68).

### Investigate the consistency of treatment effect across subgroups of clinical importance

Consistency of treatment effects across subgroups is of major importance. It impacts the interpretation of study findings and in addition affects the prescription of treatment by health care providers. Therefore, assessment of consistency is a crucial step in the interpretation of a clinical trial. In total 25 papers covered this purpose. An example illustrating consistency assessment is first described, followed by the review of the selected papers.

In a randomized double-blind clinical trial (MERIT-HF), 3991 patients with chronic heart failure (New York Heart Association (NYHA) functional class II–IV) and with ejection fraction of 0.40 or less, stabilised with optimal standard therapy, were recruited [17]. These patients were randomly assigned to either metoprolol CR/XL (*n* = 1990) (starting dose of either 12.5 mg or 25 mg once daily) or placebo (*n* = 2001). The target dose was 200 mg once daily and doses were up-titrated over 8 weeks. Dose regimen could be modified according to the judgement of the investigator. The primary endpoint was all-cause mortality. An interim analysis after a mean follow-up time of one year concluded early stopping of the trial due to established efficacy. However, in post-hoc subgroup analyses, the “all US patients” subgroup did not show a reduction in total mortality. The American regulatory agency then performed a post-hoc interaction analysis comparing “US patients” vs. “all other countries”. The hazard ratio in the former was 1.05 (95 % CI 0.71–1.56) whereas in the latter it was 0.55 (95 % CI 0.43–0.70, test for interaction *p* = 0.003). This situation invoked heated debate between the American authorities and the Investigator (MERIT-HF study group). At present, metoprolol CR/XL is indicated in the USA “for the treatment of stable, symptomatic (NYHA class II or III) heart failure of ischemic, hypertensive, or cardiomyopathic origin. It was studied in patients already receiving ACE inhibitors, diuretics, and, in the majority of cases, digitalis. In this population, metoprolol CR/XL decreased the rate of mortality plus hospitalisation, largely through a reduction in cardiovascular mortality and hospitalisations for heart failure “. The Clinical Pharmacology section of the label states that “the combined endpoints of all-cause mortality plus all-cause hospitalisation and of mortality plus heart failure hospitalisation showed consistent effects in the overall study population and the subgroups, including women and the US population. However, in the US subgroup (*n* = 1,071) and women (*n* = 898), overall mortality and cardiovascular mortality appeared less affected. Analyses of female and US patients were carried out because they each represented about 25 % of the overall population. Nonetheless, subgroup analyses can be difficult to interpret and it is not known whether these represent true differences or chance effects” [18]. This example clearly illustrates the challenges to draw inferences from (post-hoc) subgroup analyses.

Whilst “consistency” is a widely used term, there is no formal agreement about its definition [19]. As a consequence a diversity of methodological approaches exists: investigating subgroup-specific treatment effect based on the (within subgroup) statistical significance, investigating the directional treatment effect estimate in each subgroup, performing a test of interaction, or considering only qualitative interactions.

### Statistically significant consistent subgroup-specific treatment effect

Li et al. [20] investigated the probability of observing at least one statistically significant negative subgroup result conditional on a statistically significant overall positive result, when the true treatment effect is positive and homogeneous across all subgroups. In the scenario where 15 subgroups, defined by five baseline variables each defining three subgroups with equal proportions, are tested, this probability is below 1 %. Therefore, if a statistically significant negative result is observed after concluding an overall positive treatment effect, it is unlikely that this negative subgroup effect can be attributed to chance. The authors state that further investigation, such as a similar observed trend in other studies or a strong biological explanation, would be important to confirm this finding. More recently, Wang and Hung [21] investigated the probability of observing statistically significant consistent versus inconsistent treatments effects across two mutually exclusive subgroups. *Consistent* is defined as both subgroups having a statistically similar results, and *inconsistent* as the two subgroups having statistically different results. The authors show that the probability of observing statistically significant consistent effects is at most 50 %. The probability of observing a statistically significant beneficial treatment effect in both subgroups ranges from 7 % at extreme subgroup proportions to approximately 25 % at equal subgroup sample sizes. The probability that both subgroup-specific treatment effects are not shown to be statistically significant ranges from 20 % for extremely different subgroup sizes to approximately 25 % for equal subgroup sizes. Koch and Schwartz [22] proposed to apply a less stringent type I error, so that power for the subgroup test is similar to that for the overall studied population. They suggest to consider a type I error of 0.05 for subgroups with more than 70 % of the patients, 0.15 for subgroups that include about half of the patients, and 0.25 for small subgroups, e.g., ≤30 %. According to the authors, if a pre-specified subgroup fails to show any efficacy based on those criteria, the consistency of the overall treatment effect can be brought into question.

### Directionally consistent subgroup-specific treatment effect

Li et al. [20] also considered the probability of observing at least one directional negative subgroup effect conditional on a statistically significant overall positive treatment effect. Negative subgroup effect means here that the subgroup treatment effect estimate is numerically negative, under the assumption that the true treatment effect is positive and homogeneous across all subgroups. This probability is rather substantial. It is around 12 % in case 6 subgroups, defined by two baseline variables each defining three subgroups with equal proportions, are tested. When 15 subgroups are tested, defined by five baseline variables each defining three subgroups with equal proportions, the probability is around 27 %. Wang and Hung [21] also investigated the probability of observing directionally consistent versus inconsistent treatment effects: *consistent* if both subgroups have the same sign for the treatment estimate, and *inconsistent* if both subgroups have opposite sign. The probability of observing directionally consistent beneficial subgroup effects is at least 75 %. Although a homogeneous effect is expected in both subgroups, the probability of observing a numerically harmful treatment effect in one subgroup increases as the proportion of subjects in this subgroup decreases, reaching a maximum of about 25 %. Recent papers have proposed that the effect size in each subgroup must at least be either positive or some pre-defined percentage of the overall effect [23]. Keene and Garrett argued that this is not a useful approach as it fails to take into account the variability around a point estimate. Moreover, this rule might discriminate against drugs with large mean effects where large interactions are more likely. Koch and Schwartz [22] also considered this approach more subjective regardless of the plausibility. Following Hemmings [19], interpreting subgroup analyses based on point estimates alone can be misleading and provide an incomplete basis for decision making.

### Test of interaction

To assess consistency of the treatment effect across subgroups, a statistical model including interaction term(s) is commonly used. However, clinical trials are rarely powered to detect statistically significant interactions; hence results of such tests might provide a false sense of consistency [21, 23, 24]. Furthermore, the utility of the interaction test as guidance for treatment decisions is limited, as discussed recently [22, 25]. While testing for interaction is not sufficient on its own, the concept of interaction can serve as a starting point for investigating the consistency of treatment effects across subgroups. Wang and Hung [21] introduced the so-called interaction-to-overall effects ratio as the ratio of the treatment effect difference between a subgroup and its complement, and the treatment effect in the overall sample. This ratio permits the determination of the necessary sample size increase needed to detect the interaction effect. For example, when this ratio equals 2, i.e., the interaction effect is twice the overall effect, the sample size is generally large enough to detect the interaction effect. Based on this ratio, the authors provided decision rules for scenarios with two mutually exclusive subgroups. Royston and Sauerbrei [26, 27] estimated the significance level as well as the power of 21 methods for investigating interactions between treatment and a continuous covariate. Based on simulation studies the authors recommended the use of the multivariable fractional polynomial interaction procedures when dealing with continuous covariates. White and Elbourne [28] pointed out that the presence of interaction for binary data depends on the effect measure chosen (relative risk, risk difference, odds ratio). Theoretically, if a treatment effect truly exists, homogeneity in one measure, e.g., relative risk, requires heterogeneity in at least one other, e.g., risk difference. Thus the measurement scale of the interaction test, if performed, should be pre-specified and based on clinical knowledge.

### Detecting qualitative interactions

Qualitative interactions occur when one treatment is superior for one subgroup of patients and the alternative treatment is superior for the complementary subgroup [29]. True qualitative interactions are obviously of interest when one assesses the consistency of the treatment benefit across all subgroups. Several papers focus on the investigation of qualitative interactions in clinical trials. The likelihood ratio test and the range test are frequently applied in this situation. They are known to be biased and not very powerful [30, 31]. Li and Chan [32] proposed improvements to the range test. Their “extended range test” considers all observed treatment differences in a stepwise manner, instead of only taking into account the maximum and the minimum value of standardised treatment differences. Gunter et al. [33] investigated qualitative interaction, using a combination of bootstrap sampling followed by the construction of a permutation threshold. To deal with testing a large number of variables in a post-hoc manner, they proposed a variable selection technique based on the Lasso method. Kitsche and Hothorn [34] proposed a method to detect qualitative interactions for normally distributed outcome variables. They suggested using the ratio of treatment effects, which allows for the distinction between quantitative and qualitative interactions via the sign of the ratios. It allows detecting the existence of a qualitative interaction, but also estimates the magnitude of the interaction, i.e., to assess its clinical relevance. More recently, Kitsche [35] extended his method to binary outcomes using the ratio of risk differences. The author noted that the major advantage of this method is the additional information on the source and the amount of the qualitative interaction, rather than its gain in power.

### Forest plots

Forest plots are a popular graphical approach for displaying the results of subgroup analyses. As subgroup effect estimates from different baseline covariates, e.g., gender and age, may not be independent, forest plots could provide an exaggerated impression of consistency within a trial [19]. Varadhan and Wang [36] discussed a method, called standardization or inverse probability weighting, which is commonly used in epidemiology. The principle is to remove confounding due to correlation between variables by using appropriate weights. The distributions of the variables of interest become identical and therefore provide a proper unconfounded comparison of treatment effects among strata of a baseline subgrouping variable. This method can remove the bias in the estimation of subgroup-specific treatment effects as well as in the estimation of the interaction effect between the variable of interest and the treatment. However, the variance of the subgroup-specific treatment effects is increased. The authors finally advised that RCTs should report standardized subgroup-specific treatment effects with corresponding forest plots when the important baseline covariates are strongly correlated.

### Bayesian approaches

White et al. [37] stated that they were “unaware of any previous trial in which expert prior beliefs about interactions have been elicited prospectively and used to inform the interpretation of the trial”. They proposed a Bayesian approach where prior beliefs (questionnaires sent to experts) were taken into account. The idea is to use non-informative priors for the main effects, i.e., not using expert prior beliefs, and use expert beliefs only for interaction effects; hence avoiding a false positive conclusion of the trial. Bayman et al. [38] proposed a Bayesian method to detect qualitative interaction using the Bayes factor. The subgroups should be exchangeable, meaning that in the absence of qualitative interaction the treatment effect is similar across subgroups. Prior distributions for the mean and the standard deviation of the subgroups are required. Finally, the null hypothesis of no qualitative interaction is rejected when the Bayes factor is less than a pre-specified value. Jones et al. [39] challenged the idea of hypothesis testing for exploratory subgroups. They considered testing more appropriate for decision-making, and proposed shrinkage estimation techniques for exploratory post-hoc subgroup analyses. The authors applied seven different subgroup models based on a literature review, all incorporating shrinkage estimation techniques. These models are compared using the Deviance Information Criterion (DIC) and posterior predictive *p*-values. Shrinkage methods have recently drawn researchers’ attention such as Lipsky et al. [40]. Keene and Garett [23] defined shrinkage estimators as a promising approach to modeling subgroup effects, as they balance the observed subgroup treatment effects with the overall effect. Hemmings [19] also pointed this out as potentially interesting and indicated the need for research to develop this technique further. A summary of the Bayesian methodology from a more general perspective is also given by Alosh and co-workers [41].

### Other potential methods

Risk-stratification modelling may overcome some of the limitations of subgroup analyses [42–44]. The study population is stratified via a multivariable risk prediction model, taking into account multiple patient attributes (risk factors), instead of only one in subgroup-specific analyses. Each subject is thus assigned its predicted treatment effect providing a net benefit or harm. Kent et al. [44] provided two main recommendations: (1) reporting the distribution of the baseline predicted risk (or risk score) in the study population overall and by treatment arm, and if there is heterogeneity, risk stratified analysis should be performed, and (2) reporting relative and absolute risk reduction in a risk-stratified analysis. Kovalchik et al. [45] proposed a framework similar to risk-based assessment of treatment effect heterogeneity. The heterogeneity is assessed through a proportional interactions model when multiple variables influence treatment response. These covariates should be well-chosen to benefit from the statistical advantages of this method. Additionally, they provided a method for covariate selection to address model misspecification. Alosh and Huque [46] recommended the use of a pre-specified consistency criterion for a pre-specified subgroup. This criterion could be based on clinical considerations, e.g., adverse events and/or toxicities of the investigated treatment. Alosh and co-workers [47] also compared both methods with a traditional test of interaction. The main focus is on the probability of wrongly prescribing the new treatment to the subgroup that would benefit least from it. They concluded that the method from Koch and Schwarz followed by the method from Alosh and Huque could be applied to provide a flexible approach to assess safety and efficacy. Recently, Alosh et al. [41] proposed a more general approach to guide determination of the population for treatment use that takes into account the relative size of the subgroup as well as safety considerations.

### Explore the treatment effect across different subgroups within an overall non-significant trial

In case a trial for a new treatment demonstrates an overall non-significant result, treatment effects might still not be homogeneously absent across the study population. Therefore, exploring the treatment effect across different subgroups is of interest as a relevant subpopulation might benefit from that treatment [48]. The overall type I error could be substantially inflated. As a result additional confirmatory tests cannot be performed [16, 49]. Based on our search strategy, four papers were identified. The following example illustrates the situation.

The Clomethiazole Acute Stroke Study (CLASS) [50] investigated the use of clomethiazole versus placebo in 1353 patients diagnosed with an acute hemispheric stroke. The primary endpoint of the study was the relative functional independence (≥60 points on the Barthel Index, a questionnaire about activities of daily live) at 90 days. The dichotomous outcome was: functional independence or no functional independence. No significant difference was found in the relative functional independence at 90 days between the clomethiazole and placebo group (*p* = 0.649). Although no overall significant result was found, a significant interaction was observed between TACS (total anterior circulation syndrome present or not) and the treatment (clomethiazole/placebo) (*p* = 0.038). Following the interaction test, a subgroup test was conducted. Within the subgroup of TACS patients a significant difference was found between the clomethiazole and placebo group (*p* = 0.008): 40.8 % in the clomethiazole group reached relative functional independence, compared with 29.8 % in the placebo group. For the non-TACS patients no significant difference (67.3 % vs. 70.3 %) was observed (*p* = 0.358).

Tanniou et al. [48] investigated the statistical level of evidence of a single pre-specified subgroup finding in an overall statistically non-significant study with a continuous outcome. In case of a single trial, the inflation of the overall type I error is substantial and can be up to twice as large as the pre-specified value, especially in relatively small subgroups. Based on their results they defined a threshold significance level (*p*-value ≤ 0.004) for the subgroup test. They proposed to use this threshold to decide on a new replication trial, if not yet available. They also showed, unexpectedly, that testing a subgroup when there is a so-called “trend” for effect in the overall population, defined as a one-sided p-value for the overall test between 0.025 and 0.05, is bad practice with substantial overall type I error inflation up to 0.23 (one-sided). When promising subgroup findings arise in an overall non-significant trial, particular attention should be given to plausibility and replication. When a result is replicated, it often reassures decision makers [19]. In absence of other evidence, replication of promising subgroup findings should be the standard approach if the trial is overall non-significant [49]. Tanniou et al. [48] extended their research to scenarios with replications. The level of evidence is substantially improved when the subgroup finding is replicated in an independent trial. The power of the replicated trials strongly depends on the chosen replication scenario. The replication trial should preferably be planned with the same targeted effect size as in the first trial, rather than with the observed subgroup effect. The observed subgroup effect is likely to over-estimate the true subgroup effect resulting in an underpowered replication trial even though the overall type I error is almost controlled. Tanniou et al. [48] as well as Koch and Framke [49] pointed out that in case two non-significant trials reach the same significant conclusion for one particular subgroup it may form a basis to confirm efficacy of the new treatment in that subpopulation. In exceptional situations (no replication possible, urgent medical need), a case-by-case decision might lead to a positive conclusion for a promising subgroup in an overall non-significant trial. The underlying principles usually include: a clear pharmacological rationale, external evidence, a small p-value in the subgroup, and no imbalances regarding important prognostic factors present [49].

### Evaluate safety profiles limited to one or few subgroups

The size of a trial is by design expected to provide sufficient power for confirmatory comparative evidence on the primary efficacy endpoint. For safety this is often not the case. For example, there might be insufficient follow-up time for long term safety events or a limited number of observable serious safety outcomes such as deaths. As safety issues cannot be ignored, effort is needed to more effectively identify harmful effects associated with new interventions. Subgroup analyses may help identify susceptible patient groups, for which benefit-risk might be negative. Concerning the safety purpose, five papers were identified. First, an example is provided.

Tysabri (natalizumab) is used to treat adults with relapsing remitting multiple sclerosis. Its indication is restricted to highly active multiple sclerosis forms of disease due to safety concerns. Hemmings [19] provided extracts from the European Public Assessment Report (EPAR):

“Efficacy has convincingly been demonstrated at the pre-specified 2-year clinical endpoints, including the clinically highly relevant impact on disability progression, which is the major goal of treatment of relapsing remitting multiple sclerosis (MS). Treatment with 300 mg natalizumab every 4 weeks resulted in a highly statistically significant 42 % decrease in the risk of disability progression, as measured by sustained changes on Expanded Disability Status Scale, when compared to placebo over a 2-year period, and a 68 % decrease in the annualized relapse rate versus placebo over both 1 and 2 years […].

Reduced lymphocyte surveillance as induced by alpha4-integrin antagonism by natalizumab might have been causative to the occurrence of 2 cases of progressive multifocal leukencephalopathy in patients with MS, and a further case in a patient with Crohn’s disease. Two of the cases were fatal. The current safety database does not yet allow for a clear estimation of the risk of serious and/or fatal adverse events, like progressive multifocal leukoencephalopathy or other serious infections. Since MS is a disease of a typically young patient population with usually no reduced lifespan, and since there are therapeutic alternatives with an established safety profile, Tysabri should be clearly restricted to patients that are really in need of such a therapy. Expert view, also from Patient Representatives, agreed to this principle.”

In this example, there is a suspicion that Tysabri might be negative for a subgroup. There is, however, not enough data to identify the subgroup.

Koch and Framke [49] stated that if safety concerns arise, meticulous investigation should be mandatory in the assessment of a clinical trial. On a more dedicated-to-safety viewpoint, Grouin et al. [16] provided definitions and methodology about safety assessment. Subgroup analyses assessing safety differ from those assessing efficacy in the sense that their definitions are generally derived from a clinical consensus. They are usually defined by prognostic factors for safety risks, such as older age. Moreover, in case the treatment under investigation is in a market authorization process, these subgroup analyses are often demanded by authorities; hence their pre-specification is generally not necessary. The sample size for safety subgroup analyses is rather based on clinical than statistical reasons. Koch and Schwarz [22], for their part, proposed to take into account any expected safety issues into the consistency assessment. They considered the scenario where the effect size in each subgroup must at least be some percentage of the overall effect, e.g., 50 %. If any safety issue is expected, this threshold could be more conservative, e.g., 70 %, to assure a good balance between the efficacy and the safety profiles of a new treatment. The same idea holds for confirmatory strategies where a larger sample size and/or a more rigorous observed treatment effect in the complementary subgroup might be necessary to take safety issues into account.

Michiels et al. [51] investigated quantitative treatment-by-biomarker interaction in randomized controlled trials with a survival endpoint among multiple pre-defined candidate biomarkers. The authors proposed five different permutation test statistics and argued that these tests could also determine whether a subgroup of patients has a different safety profile. Similarly the multivariate risk-stratified analyses discussed above can be applied [42–44]. The authors argued that multivariable risk-stratified analyses should be conducted routinely whenever an adequate externally-developed and validated prediction tool is available. If not, safety problems occurring in low-risk subjects could be missed.

The methods from Alosh et al. and Koch and Schwartz [22, 41, 46, 47] can take into account safety issues to relax or restrict some pre-specified thresholds to ensure that any safety issue is counter-balanced by the potential efficacy of the new treatment. A further reasonable approach for assessing a safety signal for a subgroup is by pooling data from similar trials through meta-analyses. Careful consideration should then be paid to the similarity of the trials and representation of the subgroups [41].

### Establish efficacy in the targeted subgroup when included in a confirmatory testing strategy of a single trial

In the development of personalized medicine and targeted therapies it is logical to include one or more subgroups in the confirmatory testing strategy of a single trial. With the appropriate testing strategy, a significant positive subgroup finding can be considered confirmatory. Depending on the testing strategy, this can hold even if the overall test does not reach statistical significance. In any case, it provides more targeted evidence about the treatment under investigation. These study designs are to date almost exclusively applied to new oncology drugs, where both a biomarker-positive and a biomarker-negative subgroup are identified. Historically, these analyses are not common for phase III clinical trials [19]. Our intention was not to thoroughly review these biomarker-defined subgroups and associated designs, which face very specific issues. The list of papers we reviewed that address this topic is thus certainly non-exhaustive. As the pre-planned subgroup is part of the trial confirmatory testing strategy, the key methodological problem relates to the control of the type I error in multiple testing. Fridlyand et al. [52] provided an industry perspective on these designs as part of drug development and proposed decision criteria for the choice of a Phase III pivotal trial based on a Phase II result. Based on the favourable or unfavourable outcomes in all patients and/or the biomarker-based subgroup in proof-of-concept trials, different design options apply for the pivotal Phase III trial: targeted (or enrichment) design, biomarker-stratified design, traditional design, or stop the development of the new treatment. We first describe the targeted designs, then different biomarker-stratified designs, i.e., sequential subgroup-specific designs, marker sequential test designs and fallback designs, and finally other confirmatory methods are presented.

### Targeted designs

The new treatment is only evaluated in the subgroup of interest. Patients are first screened by their biomarker status and then included in the trial; hence conclusions are restricted to this subpopulation only. This design does not require any particular method to control the type I error rate [22]. It can reduce the number of randomised patients needed relative to a traditional trial design, because of the expected larger treatment effect and/or reduced variability. As the complementary subgroup is not tested, prior evidence that the treatment is very likely to work only for the subgroup of interest has to be compelling. Moreover, if the treatment effect size in the complementary subgroup is under-estimated, the potential gain associated with the targeted trial may be lost. In case of a biomarker defined subgroup, the potential benefit is also dependent on the diagnostic procedure: the higher the specificity, the better the gain in the number of randomised patients for this design [53, 54]. In some cases a targeted design might also have to enrol patients from the complementary subgroup, possibly because membership or not in the subgroup of interest requires a diagnostic procedure of which the outcome might be unknown until after the start of the randomised treatment [22]. Moreover, a diagnostic procedure never has optimal sensitivity and specificity; hence it is likely that also patients from the complementary subgroup could be included and receive a treatment. Further research has been dedicated to this topic with a more decision-theoretic approach [55].

### Biomarker-stratified designs

To investigate the effectiveness of a new treatment in a broader population, *biomarker-stratified designs* enrol both the biomarker-positive and the biomarker-negative patients. These designs may be used either to demonstrate the efficacy in the full population only or to demonstrate the efficacy in the full population or in the subgroup of interest. Multiplicity adjustments must therefore be considered to control the overall type I error rate. However, different designs might be considered depending on the prior evidence on the biomarker.

### Sequential subgroup-specific designs

In the sequential subgroup-specific design, the biomarker-positive subgroup is tested first, using a threshold of significance, e.g., 5 % (two-sided). Only if the test is statistically significant, the biomarker-negative subgroup is tested at the same significance level of 5 %. Its main advantage is that it provides clear evidence of the treatment benefit in both biomarker subgroups. However, its power is not optimal when the treatment effect is homogeneous across both subgroups [56, 57].

### Marker sequential test designs

An alternative design is the Marker Sequential Test (MaST) design. This design incorporates testings of the biomarker-positive subgroup, biomarker-negative subgroup as well as the overall studied sample. First, the biomarker-positive subgroup is tested at a reduced significance level. If the result of this test is significant, the biomarker-negative subgroup is then tested at the full significance level. If the result of the biomarker-positive subgroup is not significant, the overall studied sample is tested at a reduced significance level. This design also has the advantage to provide clear evidence about the treatment effect in both subgroups. Contrary to the sequential subgroup-specific design, it has a good statistical power when the treatment effect is homogeneous across both subgroups. However, it requires a marginally larger sample size [58].

### Fallback designs

When prior evidence on the biomarker is less convincing, a fallback design might be applied. In this two-stage design, the first stage tests the overall sample at a reduced alpha level. If the overall test is significant at that reduced level, then the trial stops and the treatment would be recommended for all patients. Otherwise the subgroup test is performed in the second stage. Wang et al. [59] indicated that the properties of this design could be improved by taking into account the correlation between the two test statistics. Spiessens and Debois [60] tackled this aspect and took into account the correlation between both tests statistics to calculate adjusted significance levels. Song and Chi [61] proposed a two-stage closed testing procedure which can be seen as an extension of the fallback procedure. A degree of efficacy consistency may be maintained by prohibiting subsequent subgroup testing if the overall test does not show any marginal significance, e.g., 0.025 < *p* < 0.10 (one-sided). This degree of consistency could be useful to avoid specific regulatory or ethical concerns. Other design parameters can be included to consider efficacy consistency requirements, such as performing the subgroup test only when there is no statistically significant quantitative interaction, or when the result of the complementary subgroup is no worse than a pre-specified boundary. Similarly, Alosh and Huque [62] proposed a method with a certain degree of consistency. Others such as Zhao et al. [63] and Alosh et al. [46] proposed extended fallback procedures.

Fallback designs focus on two primary hypotheses of interest, namely the full sample (intent-to-treat patient population) and a subgroup of interest. The clinical question whether the treatment is effective in the complementary subgroup is not addressed. Even if both primary hypothesis tests are statistically significant, the overall result might be driven by the subgroup of interest, therefore the effectiveness of the treatment in the complementary subgroup cannot be guaranteed [19, 46, 64]. Rothmann et al. [64] explored the probability of including the complementary subgroup, given that it does not truly benefit from the new treatment. The authors conclude that to extrapolate favourable efficacy to the complementary subgroup, multiplicity is not the only issue to address, but also an adequate amount of data should be provided to obtain a reliable treatment effect estimate in that group. Alosh and Huque [62] recommended defining a threshold level of evidence on the response for the least benefited (complementary) subgroup. For instance, this threshold value might be very conservative if the treatment of interest is very toxic, or be very liberal if the treatment is very safe. The authors provided extensions of three multiplicity procedures to account for the consistency requirement on the complementary subgroup.

Interim analyses might identify a potential subpopulation of interest, if not identified previously. Chen and Beckman [65] provided a method that controls the type I error rate by optimally splitting the overall significance level.

### Other confirmatory methods

Michiels et al. [51] investigated quantitative treatment-by-biomarker interaction in randomised controlled trials with a survival endpoint among multiple pre-defined candidate biomarkers. The authors proposed five different permutation test statistics, namely composite Wald, composite difference, Sum single-Wald, Max single-Wald, Fisher single-Wald. They also pointed out that a significant treatment-modifying biomarker result will always have to be replicated using data from other phase III trials. Koch and Schwartz [22] considered a modification of Hochberg’s method for controlling the multiplicity issue. A re-randomisation method can improve its power as it allows increased significance levels for both the overall and the subgroup tests based on the correlation between the subgroup test and the overall test. They also argued that if the overall test is significant and the subgroup test is not, this latter test can still be seen as consistent with a relaxed significance level. Millen et al. [25, 66] proposed both a frequentist and a Bayesian method for assessing (1) the influence and (2) the interaction conditions in order to improve the decision-making process. The purpose is to use these conditions, along with an appropriate multiple testing procedure, to provide an inferential foundation for multi-population tailoring trials. The influence condition addresses the concern that the inference in the overall population may be unduly influenced by a highly significant subgroup treatment effect. The interaction condition is only relevant when the influence condition is met. It states that to enable an enhanced product label for the pre-defined sub-population, its treatment effect has to be meaningfully greater than for the complementary subgroup. So, the goal of this condition is to help guiding decisions for a broad indication (overall population) versus an enhanced labelling (overall population indication with a specific sub-population labelling). If both conditions are met, it means that the treatment effect in the complementary subgroup is large enough, with an even greater treatment effect in the pre-specified subgroup. Sivaganesan et al. [67] investigated the use of Bayesian model selection and a threshold on posterior model probabilities to identify the relevant subgroup(s) among pre-planned subgroups. Separate classes of models are first defined for each covariate of interest. The associated posterior probabilities are then calculated for each model, and pre-specified thresholds are used to determine whether a subgroup effect should be considered or not. Eng [68] recently proposed a new method: the randomised reverse marker strategy design. Contrary to biomarker-stratified designs, which randomise treatments stratified on marker status, this design randomises patients to either the marker-based strategy or the reverse marker strategy arm. Biomarker-positive patients receive treatment A in the marker-based strategy arm and treatment B in the reverse marker strategy arm, and biomarker-negative patients receive treatment B in the marker-based strategy arm and treatment A in the reverse marker strategy arm.

## Discussion

Patients’ and health care providers’ perspectives are of main interest in drug development. In that respect, subgroup analyses could improve health care quality and thus constitute a fundamental step in the assessment of confirmatory clinical trials. Benefit-risk conclusions may hold for the overall study population, but if benefit-risk is heterogeneous across relevant subgroups this should impact the use of new drugs. The importance of subgroup analyses is acknowledged, as illustrated by recent regulatory (draft) guidance documents [69–71]. Subgroup analyses come with well-known statistical and methodological issues. Some researchers have provided general guidance to assess the credibility of subgroup findings without necessarily considering the entire context in which these analyses are involved.

It is of crucial importance to distinguish different purposes for subgroup analyses. This will help specific design or analyses choices, and will optimize the decision-making process based on the trial. Grouin et al. [16] proposed five different purposes for subgroup analyses in the context of market authorisation, which was the starting point for this review. We adapted this framework to four distinct purposes that could directly be related to design, analysis and interpretation. To gain systematic insight in the “state of the art” and areas of research needed, we reviewed the literature in line with this “purpose based” framework.

Concerning Purpose 1 (*Investigate the consistency of treatment effect across subgroups of clinical importance*), there is clear consensus that it should always be investigated. Many methods are available but some flexibility is necessary, taking into account the specific case-by-case realities of each situation. The breadth and complexity of the potential questions regarding consistency assessments leads us to the idea that a clear conceptual basis for deciding what is a desirable level of consistency in a given trial or program is of interest. This should lead to clear operational definitions of consistency.

With regard to Purpose 2 (*Explore the treatment effect across different subgroups within an overall non-significant trial*), subgroup analyses are exploratory as their level of evidence is always too weak to reach any definite answers. The best available solution is therefore to conduct a replication trial, or replicate the subgroup analysis in an already available trial. Tanniou et al. [48] proposed a threshold p-value to be used to decide on a (new) replication trial.

Purpose 3 (*Evaluate safety profiles limited to one or few subgroups*) is often not addressed in the literature, even if the safety issues are obviously a major concern . Few methods proposed take the safety assessment into account. Therefore, more research is needed. Methods to assess efficacy and safety in a multivariate way are of interest in this context.

Finally, Purpose 4 (*Establish efficacy in the targeted subgroup when included in a confirmatory testing strategy of a single trial*) is well understood and extensively investigated. To date these methods and designs are almost exclusively applied to new oncology drugs where a specific predictive biomarker is investigated. Several solutions exist to solve the main statistical issue, i.e., to control the type I error rate. On top of that a strategy for selecting the order of statistical tests needs to be defined. This is not a purely statistical problem, and comes down to where one should place their bets, citing Hemmings [19]. A clear rationale - both statistical as well as clinical - and interpretation should guide the confirmatory testing strategy.

The scientific (clinical) plausibility of subgroup results, along with consideration of the statistical strength of evidence will inform the appropriate design and analysis approach. Thus, non-statistical considerations impact the level of evidence required for either excluding a subgroup for a new treatment, or restricting use of a treatment to a subgroup.

Our framework structured the review, but also left some new research areas out of scope. Subgroup analyses, particularly those that aim to identify certain patterns or groups, are similar to classification problems in general. Specifically, methods for recursive partitioning that also address control of the inflation of the type I error rate are worth mentioning. These methods include SIDES, SIDEScreen, Virtual Twins, Interaction Trees, Model-based recursive partitioning or STIMA [72–80]. Dusseldorp and Van Michelen [81] presented a non-recursive partitioning method (QUINT). More recently, Loh et al. [82] also introduced three new regression trees to identify subgroups with potential different treatment effects. Finally, Berger et al. [83] introduced a Bayesian approach to identify subgroup effects based on defining treatment models and baseline models using tree-based methods. We did not consider the role of randomisation in our framework. In general, the consensus is that the validity of a subgroup finding is improved when stratified for at randomisation [8–10, 16, 20, 21, 49, 61]. Kaiser [84], on the contrary, argued that even when randomization is not stratified for the subgroup of interest, the treatment group sample sizes in a pre-specified subgroup on average attain the desired allocation fraction of the study overall.

A limitation of our study is that the assignment of papers to one or more of the four purposes was subjective, and for part of the papers had to be inferred. This is largely due to the fact that papers are not specific on the purpose of the methods they address.

## Conclusions

To our knowledge, this is the first methodological and statistical literature review dedicated to subgroup analyses as a methodological challenge. Many issues regarding subgroup analyses are presented in the literature without explicit reference to specific purposes. We introduced a clear framework to guide research and application of subgroup analysis. We noticed that a clear operational definition of confirming consistency of treatment effects across subgroups is lacking, but is needed to improve methods of subgroup analyses in this setting. Furthermore, researchers developing methodology should explicitly clarify the objectives of methods in terms that can be understood from a patient’s, health care provider’s and/or regulator’s perspective. Finally, methods to explore benefit-risk systematically across subgroups require further research.

## Additional file

Additional file 1:
**Pubmed Search strategy.** (DOCX 16 kb)

## Abbreviations

BMJ: British medical journal
JAMA: Journal of the American medical association
NEMJ: New England journal of medicine
CAPRIE: Clopidogrel versus aspirin in patients at risk of ischaemic events
MI: Myocardial infarction
RRR: Relative risk reduction
NICE: National institute for health and care excellence
IQWiG: Institut für qualität und wirtschaftlichkeit im gesundheitswesen
BMC: Biomed central
CR/XL: Controlled release/extended release
NYHA: New York heart association
US: United States
MERIT-HF: Metoprolol CR/XL randomised intervention trial in congestive heart failure
ACE: Angiotensin converting enzyme
RCTs: Randomized controlled trials
CHARM: Candesartan in heart failure assessment of reduction in mortality and morbidity
DIC: Deviance information criterion
CLASS: Clomethiazole acute stroke study
TACS: Total anterior circulation syndrome
EPAR: European public assessment report
MS: Multiple sclerosis
MaST: Marker sequential test
SIDES: Subgroup identification based on differential effect search
STIMA: Simultaneous threshold interaction modeling algorithm
QUINT: Qualitative interaction trees

## References

[1] Pocock SJ, Assmann SE, Enos LE, Kasten LE (2002). Subgroup analysis, covariate adjustment and baseline comparisons in clinical trial reporting: current practice and problems. Stat. Med..

[2] Hernández AV, Boersma E, Murray GD, Habbema JD, Steyerberg EW (2006). Subgroup analyses in therapeutic cardiovascular clinical trials: are most of them misleading?. Am. Heart J..

[3] Wang R, Lagakos SW, Ware JH, Hunter DJ, Drazen JM (2007). Statistics in medicine--reporting of subgroup analyses in clinical trials. N.Engl.J.Med.

[4] Gabler NB, Duan N, Liao D, Elmore JG, Ganiats TG, Kravitz RL (2009). Dealing with heterogeneity of treatment effects: is the literature up to the challenge?. Trials.

[5] Sun X, Briel M, Busse JW, Akl EA, You JJ, Mejza F, Bala M, Diaz-Granados N, Bassler D, Mertz D, Srinathan SK, Vandvik PO, Malaga G, Alshurafa M, Dahm P, Alonso-Coello P, Heels-Ansdell DM, Bhatnagar N, Johnston BC, Wang L, Walter SD, Altman DG, Guyatt GH. Subgroup Analysis of Trials Is Rarely Easy (SATIRE): a study protocol for a systematic review to characterize the analysis, reporting, and claim of subgroup effects in randomized trials. Trials. 2009;10:101. 10.1186/1745-6215-10-101 PMC2777862 19900273

[6] Sun X, Briel M, Busse JW, You JJ, Akl EA, Mejza F, Bala MM, Bassler D, Mertz D, Diaz-Granados N, Vandvik PO, Malaga G, Srinathan SK, Dahm P, Johnston BC, Alonso-Coello P, Hassouneh B, Truong J, Dattani ND, Walter SD, Heels-Ansdell D, Bhatnagar N, Altman DG, Guyatt GH. The influence of study characteristics on reporting of subgroup analyses in randomised controlled trials: systematic review. BMJ. 2011;342:d1569. 10.1136/bmj.d1569 PMC6173170 21444636

[7] Oxman AD, Guyatt GH (1992). A consumer's guide to subgroup analyses. Ann. Intern. Med..

[8] Rothwell PM (2005). Treating individuals 2. Subgroup analysis in randomised controlled trials: importance, indications, and interpretation. Lancet.

[9] Sun X, Briel M, Busse JW, You JJ, Akl EA, Mejza F, Bala MM, Bassler D, Mertz D, Diaz-Granados N, Vandvik PO, Malaga G, Srinathan SK, Dahm P, Johnston BC, Alonso-Coello P, Hassouneh B, Walter SD, Heels-Ansdell D, Bhatnagar N, Altman DG, Guyatt GH. Credibility of claims of subgroup effects in randomised controlled trials: systematic review. BMJ. 2012;344:e1553. 10.1136/bmj.e1553 22422832

[10] Dijkman B, Kooistra B, Bhandari M (2009). How to work with a subgroup analysis. Can.J.Surg.

[11] Sun X, Heels-Ansdell D, Walter SD, Guyatt G, Sprague S, Bhandari M, Sanders D, Schemitsch E, Tornetta P, Swiontkowski M. Is a subgroup claim believable? A user's guide to subgroup analyses in the surgical literature. J Bone Joint Surg Am. 2011;93:e8. 10.2106/JBJS.I.01555 PMC3028449 21266635

[12] Sun X, Briel M, Walter SD, Guyatt GH (2010). Is a subgroup effect believable? Updating criteria to evaluate the credibility of subgroup analyses. BMJ.

[13] Hasford J, Bramlage P, Koch G, Lehmacher W, Einhaupl K, Rothwell PM (2010). Inconsistent trial assessments by the National Institute for Health and Clinical Excellence and IQWiG: standards for the performance and interpretation of subgroup analyses are needed. J. Clin. Epidemiol..

[14] Bender R, Koch A, Skipka G, Kaiser T, Lange S (2010). No inconsistent trial assessments by NICE and IQWiG: different assessment goals may lead to different assessment results regarding subgroup analyses. J. Clin. Epidemiol..

[15] Hasford J, Bramlage P, Koch G, Lehmacher W, Einhaupl K, Rothwell PM (2011). Standards for subgroup analyses are needed?--we couldn't agree more. J. Clin. Epidemiol..

[16] Grouin JM, Coste M, Lewis J (2005). Subgroup analyses in randomized clinical trials: statistical and regulatory issues. J. Biopharm. Stat..

[17] MERIT-HF Study Group (1999). Effect of metoprolol CR/XL in chronic heart failure: Metoprolol CR/XL Randomised Intervention Trial in Congestive Heart Failure (MERIT-HF). Lancet.

[18] Letter of approval. Available at: http://www.accessdata.fda.gov/drugsatfda_docs/label/2009/019962s038lbl.pdf.

[19] Hemmings R (2014). An overview of statistical and regulatory issues in the planning, analysis, and interpretation of subgroup analyses in confirmatory clinical trials. J. Biopharm. Stat..

[20] Li Z, Chuang-Stein C, Hoseyni C (2007). The probability of observing negative subgroup results when the treatment effect is positive and homogeneous across all subgroups. Drug Inf J..

[21] Wang SJ, Hung HM (2014). A regulatory perspective on essential considerations in design and analysis of subgroups when correctly classified. J. Biopharm. Stat..

[22] Koch GG, Schwartz TA (2014). An overview of statistical planning to address subgroups in confirmatory clinical trials. J. Biopharm. Stat..

[23] Keene ON, Garrett AD (2014). Subgroups: time to go back to basic statistical principles?. J. Biopharm. Stat..

[24] Brookes ST, Whitely E, Egger M, Smith GD, Mulheran PA, Peters TJ (2004). Subgroup analyses in randomized trials: risks of subgroup-specific analyses; power and sample size for the interaction test. J. Clin. Epidemiol..

[25] Millen BA, Dmitrienko A, Ruberg S, Shen L (2012). A statistical framework for decision making in confirmatory multipopulation tailoring clinical trials. Drug Inf. J..

[26] Royston P, Sauerbrei W (2013). Interaction of treatment with a continuous variable: simulation study of significance level for several methods of analysis. Stat. Med..

[27] Royston P, Sauerbrei W (2014). Interaction of treatment with a continuous variable: simulation study of power for several methods of analysis. Stat. Med..

[28] White IR, Elbourne D (2005). Assessing subgroup effects with binary data: can the use of different effect measures lead to different conclusions?. BMC.Med.Res.Methodol.

[29] Peto P, Halnan KE (1982). Statistical Aspects of Cancer Trials. Treatment of Cancer.

[30] Piantadosi S, Gail MH (1993). A comparison of the power of two tests for qualitative interactions. Stat. Med..

[31] Zelterman D (1990). On tests for qualitative interactions. Stat Probabil Lett.

[32] Li J, Chan IS (2006). Detecting qualitative interactions in clinical trials: an extension of range test. J. Biopharm. Stat..

[33] Gunter L, Zhu J, Murphy S (2011). Variable selection for qualitative interactions in personalized medicine while controlling the family-wise error rate. J. Biopharm. Stat..

[34] Kitsche A, Hothorn LA (2014). Testing for qualitative interaction using ratios of treatment differences. Stat. Med..

[35] Kitsche A (2014). Detecting qualitative interactions in clinical trials with binary responses. Pharm. Stat..

[36] Varadhan R, Wang SJ (2014). Standardization for subgroup analysis in randomized controlled trials. J. Biopharm. Stat..

[37] White IR, Pocock SJ, Wang D (2005). Eliciting and using expert opinions about influence of patient characteristics on treatment effects: a Bayesian analysis of the CHARM trials. Stat. Med..

[38] Bayman EO, Chaloner K, Cowles MK (2010). Detecting qualitative interaction: a Bayesian approach. Stat. Med..

[39] Jones HE, Ohlssen DI, Neuenschwander B, Racine A, Branson M (2011). Bayesian models for subgroup analysis in clinical trials. Clin. Trials.

[40] Lipsky AM, Gausche-Hill M, Vienna M, Lewis RJ (2010). The importance of "shrinkage" in subgroup analyses. Ann. Emerg. Med..

[41] Alosh, M., Fritsch, K., Huque, M., Mahjoob, K., Pennello, G., Rothmann, M. , Russek-Cohen, E. , Smith, F., Wilson, S. and Yue, L. (2015). Statistical considerations on subgroup analysis in clinical trials. Statistics in Biopharmaceutical Research (accepted), available on line at: http://www.tandfonline.com/doi/full/10.1080/19466315.2015.1077726

[42] Hayward RA, Kent DM, Vijan S, Hofer TP (2006). Multivariable risk prediction can greatly enhance the statistical power of clinical trial subgroup analysis. BMC.Med.Res.Methodol..

[43] Kent DM, Hayward RA (2007). Limitations of applying summary results of clinical trials to individual patients: the need for risk stratification. JAMA.

[44] Kent DM, Rothwell PM, Ioannidis JP, Altman DG, Hayward RA (2010). Assessing and reporting heterogeneity in treatment effects in clinical trials: a proposal. Trials.

[45] Kovalchik SA, Varadhan R, Weiss CO (2013). Assessing heterogeneity of treatment effect in a clinical trial with the proportional interactions model. Stat. Med..

[46] Alosh M, Huque MF (2013). Multiplicity considerations for subgroup analysis subject to consistency constraint. Biom. J..

[47] Alosh M, Huque MF, Koch GG (2014). Statistical perspectives on subgroup analysis: testing for heterogeneity and evaluating error rate for the complementary subgroup. J. Biopharm. Stat..

[48] Tanniou J, Tweel IV, Teerenstra S, Roes KC, „Level of evidence for promising subgroup findings in an overall non-significant trial,” Stat Methods Med Res, 2014 (accepted), available on line at: http://smm.sagepub.com/content/early/2014/01/19/0962280213519705.full.pdf+html. 10.1177/0962280213519705 24448444

[49] Koch A, Framke T (2014). Reliably basing conclusions on subgroups of randomized clinical trials. J. Biopharm. Stat..

[50] Wahlgren NG, Ranasinha KW, Rosolacci T, Franke CL, van Erven PM, Ashwood T, Claesson L. Clomethiazole acute stroke study (CLASS): results of a randomized, controlled trial of clomethiazole versus placebo in 1360 acute stroke patients. Stroke. 1999;30:21–8. 10.1161/01.str.30.1.21 9880383

[51] Michiels S, Potthoff RF, George SL (2011). Multiple testing of treatment-effect-modifying biomarkers in a randomized clinical trial with a survival endpoint. Stat. Med..

[52] Fridlyand J, Yeh RF, Mackey H, Bengtsson T, Delmar P, Spaniolo G, Lieberman G. An industry statistician's perspective on PHC drug development. Contemp Clin Trials. 2013;36:624–35. 10.1016/j.cct.2013.04.006 23648396

[53] Maitournam A, Simon R (2005). On the efficiency of targeted clinical trials. Stat. Med..

[54] Simon R (2010). Clinical trials for predictive medicine: new challenges and paradigms. Clin. Trials.

[55] Krisam J, Kieser M (2014). Decision rules for subgroup selection based on a predictive biomarker. J. Biopharm. Stat..

[56] Simon R (2012). Clinical trials for predictive medicine. Stat. Med..

[57] Freidlin B, Korn EL (2014). Biomarker enrichment strategies: matching trial design to biomarker credentials. Nat. Rev. Clin. Oncol..

[58] Freidlin B, Korn EL, Gray R (2014). Marker Sequential Test (MaST) design. Clin. Trials.

[59] Wang SJ, O'Neill RT, Hung HM (2007). Approaches to evaluation of treatment effect in randomized clinical trials with genomic subset. Pharm. Stat..

[60] Spiessens B, Debois M (2010). Adjusted significance levels for subgroup analyses in clinical trials. Contemp. Clin. Trials.

[61] Song Y, Chi GY (2007). A method for testing a prespecified subgroup in clinical trials. Stat. Med..

[62] Alosh M, Huque MF (2009). A flexible strategy for testing subgroups and overall population. Stat. Med..

[63] Zhao YD, Dmitrienko A, Tamura R (2010). Design and analysis considerations in clinical trials with a sensitive subpopulation. Stat. Biopharm. Res..

[64] Rothmann MD, Zhang JJ, Lu L, Fleming TR (2012). Testing in a prespecified subgroup and the intent-to-treat population. Drug Inf. J..

[65] Chen C, Beckman RA (2009). Hypothesis testing in a confirmatory Phase III trial with a possible subset effect. Stat. Biopharm. Res..

[66] Millen BA, Dmitrienko A, Song G (2014). Bayesian assessment of the influence and interaction conditions in multipopulation tailoring clinical trials. J. Biopharm. Stat..

[67] Sivaganesan S, Laud PW, Muller P (2011). A Bayesian subgroup analysis with a zero-enriched Polya Urn scheme. Stat. Med..

[68] Eng KH (2014). Randomized reverse marker strategy design for prospective biomarker validation. Stat. Med..

[69] EMA/CHMP/EWP/117211/2010, Committee for Medicinal Products for Human Use CHMP. Concept paper on the need for a Guideline on the use of Subgroup Analyses in Randomised Controlled Trials. 2010. http://www.ema.europa.eu/docs/en_GB/document_library/Scientific_guideline/2010/05/WC500090116.pdf.

[70] Food and Drug Administration. Enrichment Strategies for Clinical Trials to Support Approval of Human Drugs and Biological Products. 2012. http://www.fda.gov/downloads/drugs/guidancecomplianceregulatoryinformation/guidances/ucm332181.pdf.

[71] EMA/CHMP/539146/2013, Committee for Medicinal Products for Human Use CHMP. Guideline on the investigation of subgroups in confirmatory clinical trials. 2014. http://www.ema.europa.eu/docs/en_GB/document_library/Scientific_guideline/2014/02/WC500160523.pdf.

[72] Lipkovich I, Dmitrienko A, Denne J, Enas G (2011). Subgroup identification based on differential effect search--a recursive partitioning method for establishing response to treatment in patient subpopulations. Stat. Med..

[73] Lipkovich I, Dmitrienko A (2014). Strategies for identifying predictive biomarkers and subgroups with enhanced treatment effect in clinical trials using SIDES. J.Biopharm.Stat.

[74] Foster JC, Taylor JM, Ruberg SJ (2011). Subgroup identification from randomized clinical trial data. Stat. Med..

[75] Su X, Zhou T, Yan X, Fan J, Yang S. Interaction trees with censored survival data. Int J Biostat. 2008;4. 10.2202/1557-4679.1071 PMC2835451 20231911

[76] Su X, Tsai CL, Wang H, Nickerson DM, Li B (2009). Subgroup analysis via recursive partitioning. J. Mach. Learn. Res..

[77] Doove LL, Dusseldorp E, Van DK, Van M I (2014). A comparison of five recursive partitioning methods to find person subgroups involved in meaningful treatment–subgroup interactions. Adv. Data Anal. Classif..

[78] Zeileis A, Hothorn T, Hornik K (2008). Model-based recursive partitioning. J. Comput. Graph. Stat..

[79] Dusseldorp E, Conversano C, Van Os BJ (2010). Combining an additive and tree-based regression model simultaneously: STIMA. J. Comput. Graph. Stat..

[80] Ruberg SJ, Chen L, Wang Y (2010). The mean does not mean as much anymore: finding sub-groups for tailored therapeutics. Clin. Trials.

[81] Dusseldorp E, Van M I (2014). Qualitative interaction trees: a tool to identify qualitative treatment-subgroup interactions. Stat. Med.

[82] Loh WY, He X, Man M (2015). A regression tree approach to identifying subgroups with differential treatment effects. Stat. Med..

[83] Berger JO, Wang X, Shen L (2014). A Bayesian approach to subgroup identification. J. Biopharm. Stat..

[84] Kaiser LD (2013). Stratification of randomization is not required for a pre-specified subgroup analysis. Pharm. Stat..

