# Supplementary material for: Subgroup analyses in confirmatory clinical trials: time to be specific about their purposes
Source: BMC Med Res Methodol. 2016 Feb 18;16:20. doi: 10.1186/s12874-016-0122-6 (PMC4757983; doi:10.1186/s12874-016-0122-6)
Supplement: Additional file 1: — Pubmed Search strategy. (DOCX 16 kb) [file 12874_2016_122_MOESM1_ESM.docx]

(subpopulation[Title/Abstract] OR

subpopulations[Title/Abstract] OR

sub-population[Title/Abstract] OR

sub-populations[Title/Abstract] OR

subset[Title/Abstract] OR

subsets[Title/Abstract] OR

sub-set[Title/Abstract] OR

sub-sets[Title/Abstract] OR

subgroup[Title/Abstract] OR

subgroups[Title/Abstract] OR

sub-group[Title/Abstract] OR

sub-groups[Title/Abstract] OR

interaction[Title/Abstract] OR

interactions[Title/Abstract] OR

heterogeneity[Title/Abstract] OR

heterogeneous[Title/Abstract] OR

confounding[Title/Abstract] OR

confoundings[Title/Abstract] OR

forest[Title/Abstract])

AND

(Journal of biopharmaceutical statistics[Journal] OR Statistics in medicine[Journal] OR Contemporary clinical trials[Journal] OR Trials[Journal] OR Clinical trials[Journal] OR Pharmaceutical statistics[Journal] OR BMC Medical Research Methodology[Journal] OR Drug information journal[Journal] OR Statistical methods in medical research[Journal] OR Biostatistics[Journal] OR [Biometrical Journal](http://www.interscience.wiley.com/jpages/0323-3847/)[Journal] OR [Biometrika](http://www.biomet.oupjournals.org)[Journal] OR [Statistical Methodology](http://www.elsevier.com/locate/stamet)[Journal] OR Biometrics[Journal])

AND

("2005"[PDAT] : "2015/05/01"[PDAT])

Caption: Pubmed Search strategy
